# Factors associated with death and loss to follow-up in children on antiretroviral care in Mingalardon Specialist Hospital, Myanmar, 2006–2016

**DOI:** 10.1371/journal.pone.0195435

**Published:** 2018-04-05

**Authors:** Kay Khaing Kaung Nyunt, Wai Wai Han, Srinath Satyanarayana, Petros Isaakidis, San Hone, Aye Aye Khaing, Hoa Nguyen Binh, Htun Nyunt Oo

**Affiliations:** 1 National AIDS Programme, Department of Public Health, Ministry of Health and Sports, Nay Pyi Taw, Myanmar; 2 Department of Medical Research, Ministry of Health and Sports, Yangon, Myanmar; 3 Center for Operational Research, International Union Against Tuberculosis and Lung Disease, Paris, France; 4 Médecins Sans Frontières (MSF)/Doctors Without Borders, Mumbai, India; 5 Médecins Sans Frontières (MSF)/Doctors Without Borders, Operational Research Unit, Luxembourg; 6 Mingaladon Specialist Hospital, Department of Medical Care, Ministry of Health and Sports, Yangon, Myanmar; 7 National Tuberculosis Program Vietnam, Hanoi, Viet Nam; Agencia de Salut Publica de Barcelona, SPAIN

## Abstract

**Background:**

Myanmar National AIDS programme’s priority is to improve the survival of all people living with HIV by providing anti-retroviral therapy (ART) care. More than 7200 children (aged <15 years) have been enrolled into ART care from 2005 to 2016. A previous study showed that ~11% children on ART care had either died or were lost to follow-up by 60 months. Factors associated with death and lost-to follow-up (adverse outcomes) have not been previously studied.

**Objectives:**

To describe the association between demographic and clinical characteristics at enrollment into ART care with adverse outcomes.

**Methods:**

Cohort study using records of children enrolled for ART care at Mingalardon Specialist Hospital (main Paediatric ART center in Myanmar) from 2006–2016. We used multivariable Cox proportional hazards regression models for analysis.

**Results:**

1,159 children were enrolled for ART care and they contributed a total of 1.45 million person-days of follow-up period. 112 (10%) had an adverse outcome during the follow-up time period (55 deaths, 57 lost to follow-up). Enrollment into the ART care through in-patient care department of the hospital, CD4 Cell count <50/mm^3^, enrollment during changing ART guidelines (different ART eligibility criteria and preferred ART regimen) were independently associated with higher hazards of adverse outcome. Receiving protease inhibitor-based ART regimen at enrollment was independently associated with lower hazards of adverse outcome. Age, sex, residing in urban or rural areas, WHO clinical stage, having TB at the time of enrollment, receiving cotrimoxazole prophylaxis were not statistically associated with adverse outcomes.

**Conclusion:**

Our analysis reconfirms good survival of children on ART care (including those with TB). The characteristics associated with adverse outcomes (other than CD4 cell count<50) are surrogates of some unmeasured underlying health system/ patient related factors that needs further exploration to improve the survival of children on ART care.

## Background

Globally 36.7 million people are living with HIV (PLHIV) and of these 5.1 million are in Asia and Pacific Region of the World Health Organisation. There were ~19 000 [16 000–22 000] new HIV infections in children in this region in 2015[1]. By 2016, approximately 127,402 people were enrolled in HIV care in Myanmar and this includes 7,298 children below the age of 15 years [2]. Antiretroviral treatment (ART) services have gradually expanded throughout the country. Currently 269 health facilities are providing ART care nationwide (219 public sector and 50 NGO/INGO/private sector sites) with the public sector providing ART to 62% of the total PLHIV in the country[3]. The first pediatric ART center was initiated in the public sector in 2005 at Mingalardon Specialist Hospital, Yangon.

Evidence suggests that early HIV diagnosis and ART can reduce mortality by 76% and HIV progression by 75% in HIV infected children [4]. However, ART outcomes among children in Myanmar have not been adequately studied and reported. A PubMed search revealed several pediatric HIV studies from the South East Asia region but none from Myanmar. To our knowledge, there has been only one locally published report describing the mortality/survival of pediatric HIV cohort in the country[5]. This study looked at deaths among children enrolled for ART care from 2005 to 2012 in Mingalardon Specialist Hospital, Yangon and Mandalay Children hospital and reported the cumulative survival probabilities at various time periods. The 12 month and 60 month cumulative survival probabilities were 0.92 and 0.89 respectively[5]. However, this study did not report on the factors associated with adverse treatment outcomes (death and loss to follow-up). Identifying the factors associated with adverse outcomes will help in informing the policies and practices for further improving the survival of children on ART care in the country.

Therefore, the objectives of this study are, a) to describe the demographic and clinical characteristics of the children enrolled for ART care at Mingalardon Specialist Hospital, Yangon from 2006–2016, and b) to assess the association between these demographic and clinical characteristics with adverse treatment outcomes (death and loss to follow-up).

## Methods

### Design

This was a retrospective cohort study using records of children (aged < 15 years) enrolled for ART care at Mingalardon Specialist Hospital during the period 2006 to 2016.

### Study setting

#### General setting

Myanmar is the largest country in mainland South East Asia with a total land area of 676,578 square kilometers. According to the 2014 Population and Housing Census, 51.4 million people are living in Myanmar with a population density is 76 persons per square kilometer. Approximately 70% of the population resides in rural areas[6]. The country is divided administratively, into Nay Pyi Taw Union Territory and 14 States and Regions. Myanmar health system has primary, secondary and tertiary health care facilities. All regions/states have primary and secondary health care services but tertiary health facilities are only in some large regions. In each region/state there are three to four districts. There are four to five township hospitals in each district and one to two station hospitals and six to seven rural health centres in each township.

#### Myanmar National AIDS Program (NAP)

NAP is one of the disease control programs under Disease Control Unit, Department of Public Health, Ministry of Health and Sports. It was formed in 1989 by integration with existing sexually transmitted diseases program. The major components of the programme are prevention, early diagnosis, providing ART care, monitoring and evaluation. Pediatric ART care was initiated in the public sector in 2005, and currently there are 106 paediatric ART centers. Mingalardon Specialist Hospital in Yangon is one of the main ART centers among them. At this hospital, children can get enrolled into the ART care after being referred from the out-patient department, inpatient department of the hospital, or after referral from any other health facility. Prior to enrollment the child undergoes baseline clinical investigations which includes assessment for WHO stage, CD4 Cell count and opportunistic infections (especially TB). The guidelines for initiating the child on ART were in accordance with the WHO guidelines at different time periods and were revised in 2005, 2007, 2011, 2014 **(Table 1**) [7–10]. Once enrolled, children visit this center at regular intervals for follow-up evaluations. The details of all children enrolled on ART care is being maintained in an electronic database.

**Table 1:** National Guidelines for ART eligibility for HIV seropositive children age ≤ 15 years from 2005 to 2016, Myanmar.

Guidelines	ART eligibility criteria	Preferred ART regimen
National Guidelines	• WHO Stage 4 or 3	AZT or d4T +3TC+NVP or EFV (if age >3 years)
[Guideline 2005]	• Age ≤18 months, WHO Stage 1 or 2 with TLC <2500mm^3^	
	• Age >18 months, WHO Stage 1 or 2 with TLC <1500mm^3^	
	• If CD4 cell counts are available, then	
	○ Age ≤18 months, any WHO Stage (1/2/3/4) with % CD4 <20%	
	○ Age >18 months, any WHO Stage (1/2/3/4) with % CD4 <15%	
National Guidelines	• WHO Stage 3 or 4	AZT or d4T +3TC+NVP or EFV (if age >3 years)
[Guidelines 2007]	• CD4 indicating severe HIV associated immune deficiency	
	○ Age <11 months with CD4 <25%	
	○ Age 12–35 months with CD4 <20%	
	○ 36–59 months with CD4 <15%	
	○ ≥5 years with CD4 <15% or CD4 Cell count <200 cells/mm^3^	
National Guidelines	• All HIV infected children less than 2 years	If age < 2 years
[Guidelines 2011]	• For children > 2 years, if WHO Stage 3 or 4	• AZT+3TC+NVP for NVP naïve children
	• For children > 2 years, and WHO Stage 1 or 2 if	• AZT+3TC+LPV/r for NNRTI exposed children
	○ Age 2 to 5 years and CD4 cell count ≤750 cells/mm^3^	If age 2 to 3 years—>
	○ Age >5 years and CD4 cell count ≤ 350 cells/mm^3^	• AZT or ABC or d4T+3TC+NVP
		If age > 3 years
		• AZT or ABC or d4T+3TC+NVP or EFV
National Guidelines	• All HIV infected children age ≤ 5 years	If age <3 years (any of the two regimens)
[Guideline 2014]	• For children > 5 years, if	• ABC or AZT+3TC+LPV/r
	○ CD4 cell count ≤ 500cells/mm^3^;	• ABC or AZT+3TC+NVP
	○ WHO Stage 3 or 4	If age 3 to 10 years (any of the four regimens)
	○ HIV-TB co infection	• ABC+3TC+EFV
		• ABC or AZT+3TC+NVP
		• TDF+3TC+NVP or EFV
		• AZT+3TC+EFV
		If age 10 to 19 years (any of the 3 regimens)
		• TDF+3TC+EFV
		• AZT+3TC+EFV or NVP
		• TDF+3TC+NVP

ART = Anti-retroviral therapy; HIV = Human immunodeficiency virus (HIV); TLC = Total Leucocyte count; WHO = World Health Organisation; ABC = Abacavir; AZT = Zidovudine; d4t –Stavudine; EFV = Efavirenz;, LPV/r-lopinavir boosted ritonavir; NVP = Nevirapine; TDF-Tenofovir disoproxil fumarate; 3TC-lumivudine

### Study population

All HIV positive children aged <15 years who were enrolled for ART care at Mingalardon Specialist Hospital for pediatric ART care between 1^st^January 2006 to 31^st^ December 2016.

### Definitions of outcomes

Each child enrolled into our study was assigned one of these four mutually exclusive outcomes as on 31^st^ March, 2017 (date of censoring).

Alive and on ART care:—Patients who are alive and continue to receive ART care from the ART center.

Death:—patients who have been reported as death from family members during the ART treatment.

Lost to follow up (LTFU)–defined when the patient missing for 3 consecutive months the follow up visit.

Transfer-out:—patients who were transferred to other ART clinics for continuation of care or moved from pediatric ART care to adult ART care (at 15 years of age).

Each child’s duration in ART care was calculated from their date of enrolment into ART care to the date of their outcome or date of censoring (which ever date happened to be earlier). In our study, we defined adverse treatment outcome as any child who has died or was lost-to-follow-up. Children who were “alive and on ART care” or children who were “transferred out” were considered as favorable outcomes.

### Data variables, sources of data and data collection

Data variables included (i) socio-demographic and clinical characteristics at enrollment into ART care and (ii) treatment outcomes of the HIV infected children. We obtained individual patient data from electronic patient database of Mingalardon Specialist Hospital. The demographic and clinical factors that we assessed for its association with adverse outcomes were those that are routinely maintained in this electronic database. These included age, sex, residence in urban or rural areas, point of entry into ART care (from outpatient department/ or from in-patient care), WHO Stage, CD4 Cell count, and ART guidelines at the time of enrollment, presence or absence of TB at the time of enrollment into ART care, type of TB, ART regimen (NNRTI-based regimen or PI-based regimen) and whether the child received co-trimoxazole prophylaxis for opportunistic infection at enrollment.

### Analysis and statistics

Relevant variables from electronic database of the hospital were extracted into Excel and imported to Stata/IC version 12.1 (StataCorp LP, TX, USA) for analysis. The demographic and clinical characteristics of the study population have been described using numbers and proportions. We used unadjusted Kaplan-Meier survival methods and curves to assess the differences in the survival of children with various demographic and clinical characteristics. We used Cox proportional hazards models to estimate the hazard ratios and adjusted hazard ratios (HRs & aHRs) of the association between demographic and clinical factors and adverse treatment outcomes. We assessed for violations of the proportionality assumption using schoenfeld and scaled schoenfeld residuals (using stphtest command in Stata). The factors that were included in multivariable model to estimate adjusted hazard ratios included variables that were significantly associated at the bi-variate level or were known to be previously associated with adverse outcomes in published literature. Statistical significance was set at 0.05.

### Ethics

Ethics approval was obtained from Ethics Review Committee of Department of Medical Research, Ministry of Health and Sports, Myanmar (ERC Approval Number Ethics/DMR/2017/028) and the Ethics Advisory Group of The Union, Paris, France (86/16). The Director of Disease Control Programmes, Ministry of Health and Sports, Myanmar approved to use the patient data for this study.

## Results

From 2006 to2016, 1,159 children were enrolled for ART care. Their demographic characteristics are described in **Table 2** along with the time period of enrollment into ART care. Nearly half (53%) were males with three fourths (76%) being children in the age group 1–10 years. Majority of them (83%) were from urban areas and their main entry point into ART care was referral from out-patient departments (78%). Mother to child transmission was the predominant mode of HIV infection for almost all of them (95%).

**Table 2:** Socio-demographic characteristics of HIV positive children (<15 years of age) [N = 1159] enrolled for Antiretroviral therapy in Mingalardon Specialist Hospital in Yangon between 2006 and 2016.

Socio-demographic characteristics	Overall		2006–2009		2010–2013		2014–2016	
	Number	(%) (Column)						
			Number	% (row)	Number	% (row)	Number	% (row)
Age								
<1 years	83	7.2	2	2.4	30	36.1	51	61.4
1–5 years	397	34.2	59	14.9	149	37.5	189	47.6
5–10 years	482	41.6	78	16.2	156	32.4	248	51.5
>10–15 years	197	17	16	8.1	68	34.5	113	57.4
Sex								
Male	610	52.6	77	12.6	217	35.6	316	51.8
Female	549	47.4	78	14.2	186	33.9	285	51.9
Permanent Address								
Urban	965	83.3	154	16	356	36.9	455	47.2
Rural	194	16.7	1	0.5	47	24.2	146	75.3
Entry point of care								
Out-patient	906	78.2	132	14.6	358	39.5	416	45.9
In-patient	228	19.7	11	4.8	35	15.4	182	79.8
Others	18	1.5	10	55.6	6	33.3	2	11.1
Unknown	7	0.6	2	28.6	4	57.1	1	14.3
Risk factor of patients								
Blood transfusion	2	0.2	1	50.0	1	50.0	0	0.0
Mother to child	1100	94.9	151	13.7	365	33.2	584	53.1
Unknown	57	4.9	3	5.3	37	64.9	17	29.8

**Table 3** shows their clinical characteristics. Nearly 70% were in WHO stage 3 or stage 4. Nearly half (51%) of them had CD4 cell counts less than 350 cells/mm3 with about one fifth (17%) having CD4 count <50 cells/mm^3^. More than half of them (58%) had tuberculosis (predominantly pulmonary TB) at the time of enrollment. Cotrimoxazole prophylaxis for opportunistic infections was given to a large proportion of children at enrolled (83%). Most of them (94%) received Non-nucleoside reverse transcriptase inhibitor (NNRTI) based regimen at enrollment and majority (86%) were alive and still on ART care at this center on the date of censoring.

**Table 3:** Clinical characteristics of HIV positive children (<15 years of age) [N = 1159] at enrolment into Anti-Retroviral therapy Care (ART) care in Mingalardon Specialist Hospital in Yangon between 2006 and 2016.

Clinical characteristics	Overall		2006–2009		2010–2013		2014–2016	
	Number	% (column)	Number	% (row)	Number	% (row)	Number	% (row)
WHO Staging								
- Stage 1	231	19.9	1	0.4	11	4.8	219	94.8
- Stage 2	109	9.4	4	3.7	34	31.2	71	65.1
- Stage 3	623	53.8	111	17.8	254	40.8	258	41.4
- Stage 4	196	16.9	39	19.9	104	53.1	53	27.0
CD4 Count baseline								
- <50 cells/mm^3^	199	17.2	38	19.1	76	38.2	85	42.7
- 50–200 cells/mm^3^	246	21.2	29	11.8	99	40.2	118	48.0
- 201–350 cells/mm^3^	147	12.7	23	15.6	64	43.5	60	40.8
- 351–500 cells/mm^3^	120	10.4	19	15.8	35	29.2	66	55.0
- >500 cells/mm^3^	384	33.1	34	8.9	109	28.4	241	62.8
- Missing	63	5.4	12	19.0	20	31.7	31	49.2
TB								
- Yes	674	58.2	121	18.0	263	39.0	290	43.0
- No	485	41.8	34	7.0	140	28.9	311	64.1
TB status during ART treatment								
- Pulmonary TB (Smear +)	4	0.6	1	25.0	0	0.0	3	75.0
- Pulmonary TB (smear -)	116	17.2	60	51.7	48	41.4	8	6.9
- Extra Pulmonary TB	51	7.6	8	15.7	13	25.5	30	58.8
- Pulmonary TB	465	69	43	9.2	181	38.9	241	51.8
- Missing	38	5.6	9	23.7	21	55.3	8	21.1
Prophylaxis for OI								
- Yes	960	82.8	143	14.9	345	35.9	472	49.2
- No	199	17.2	12	13.4	58	29.1	129	64.8
Outcome of ART patients								
- Alive and on ART	993	85.7	116	11.7	341	34.3	536	54.0
- Death	57	4.9	10	17.5	29	50.9	18	31.6
- Lost to follow up	55	4.7	6	10.9	18	32.7	31	56.4
- Transfer out	54	4.7	23	42.6	15	27.8	16	29.6
Regime of ART patients								
-NNRTI based regime	1092	94.2	137	12.5	380	34.8	575	52.7
- PI based regime	67	5.8	18	26.9	23	34.3	26	38.8

NNRTI-Non-Nucleoside reverse-transcriptase inhibitor; PI-Protease inhibitor; OI-opportunistic infection; CD4-CD4 +T lymphocyte count.

The children cumulatively contributed a total of 1,453,260 child-days of follow-up time period on pediatric ART care, 54 (4%) were transferred from this center and 112 (10%) children had an unfavorable outcome (death or loss to follow-up). The overall median duration of follow-up was 1110 days (Interquartile range: 539–1974 days). The median duration of follow-up time period in children who were lost to follow-up was 111 days (Interquartile range: 42–413 days) and in those who had died was 48 days (interquartile range: 19–158 days).

**Fig 1:**
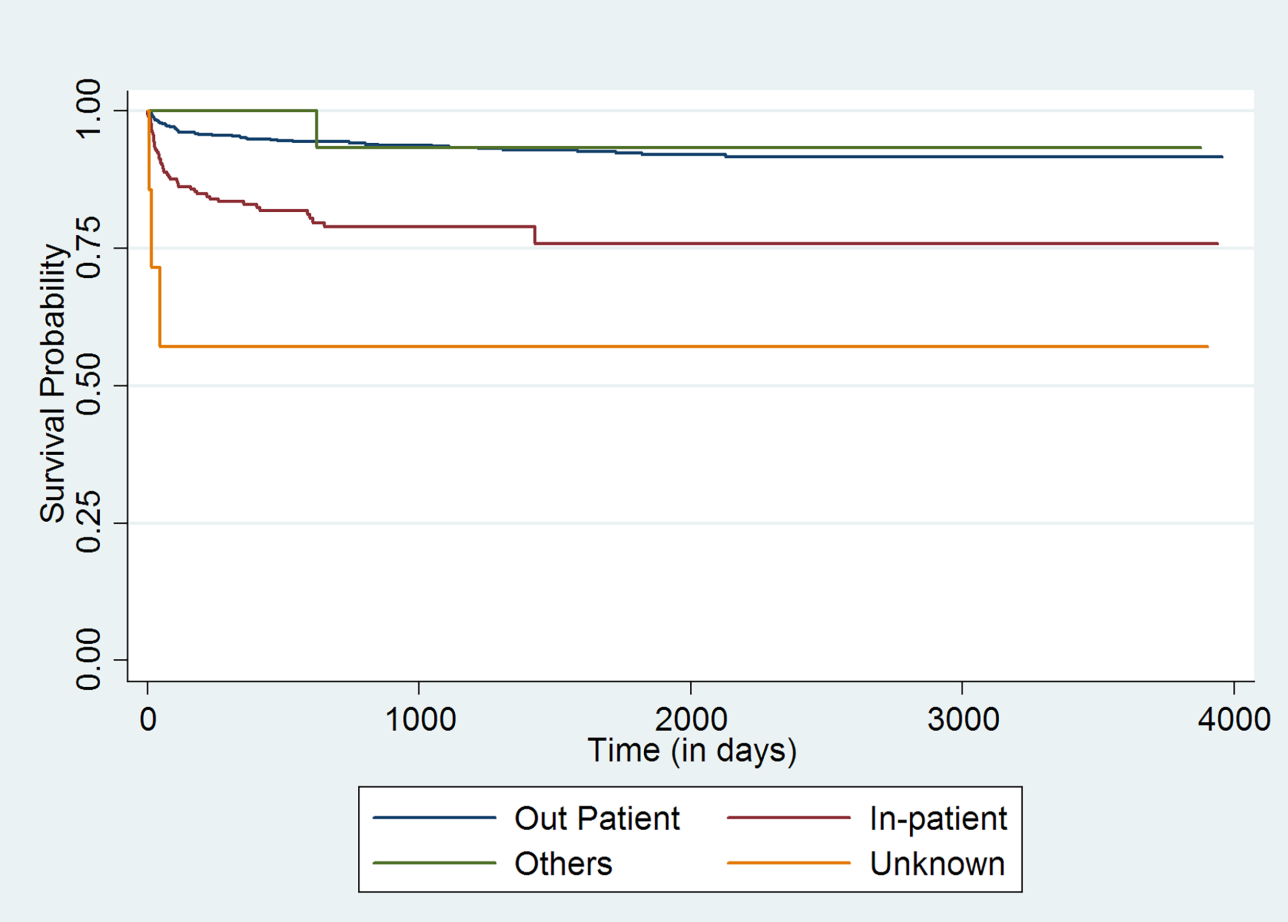
Unadjusted Kaplan-Meier survival curves for treatment outcome of HIV positive children who receiving ART in Mingalardon Specialist Hospital by entry point of care (2006 to 2016). Log rank test chi2 p-value: 0.0000.

**Fig 2:**
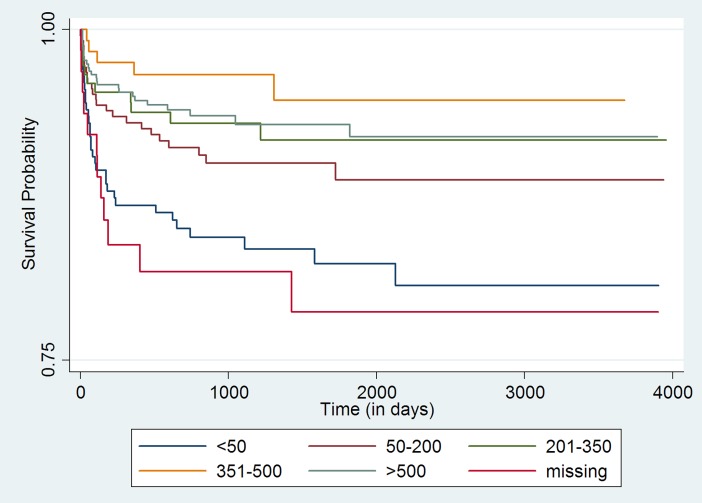
Unadjusted Kaplan-Meier survival curves for treatment outcome of HIV positive children who receiving ART in Mingalardon Specialist Hospital by CD4 count (2006 to 2016). Log rank test chi2 p-value: 0.0001.

**Fig 3:**
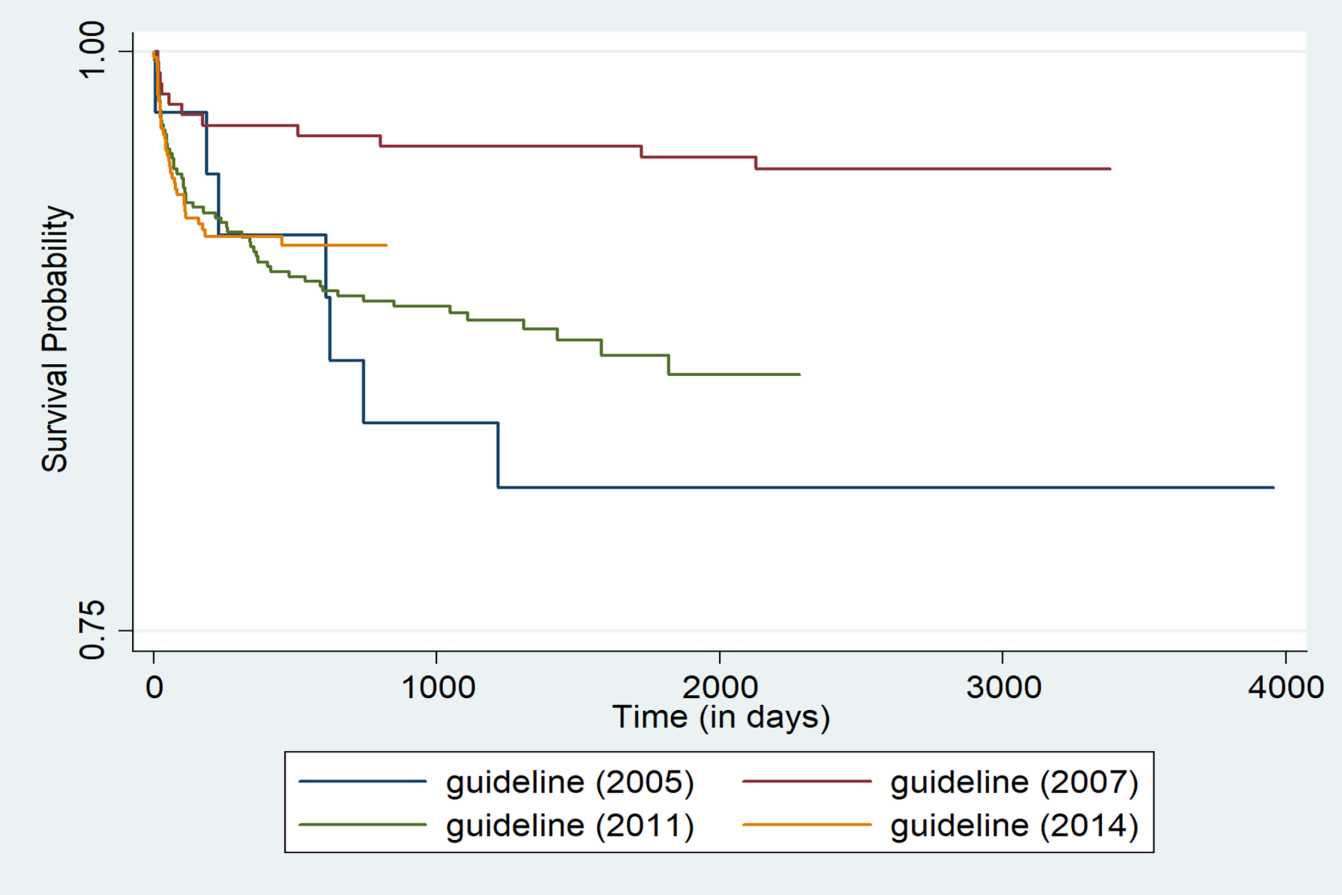
Unadjusted Kaplan-Meier survival curves for treatment outcome of HIV positive children who receiving ART in Mingalardon Specialist Hospital by guideline (2006 to 2016). Log rank test chi2 p-value: 0.0042.

**Fig 4:**
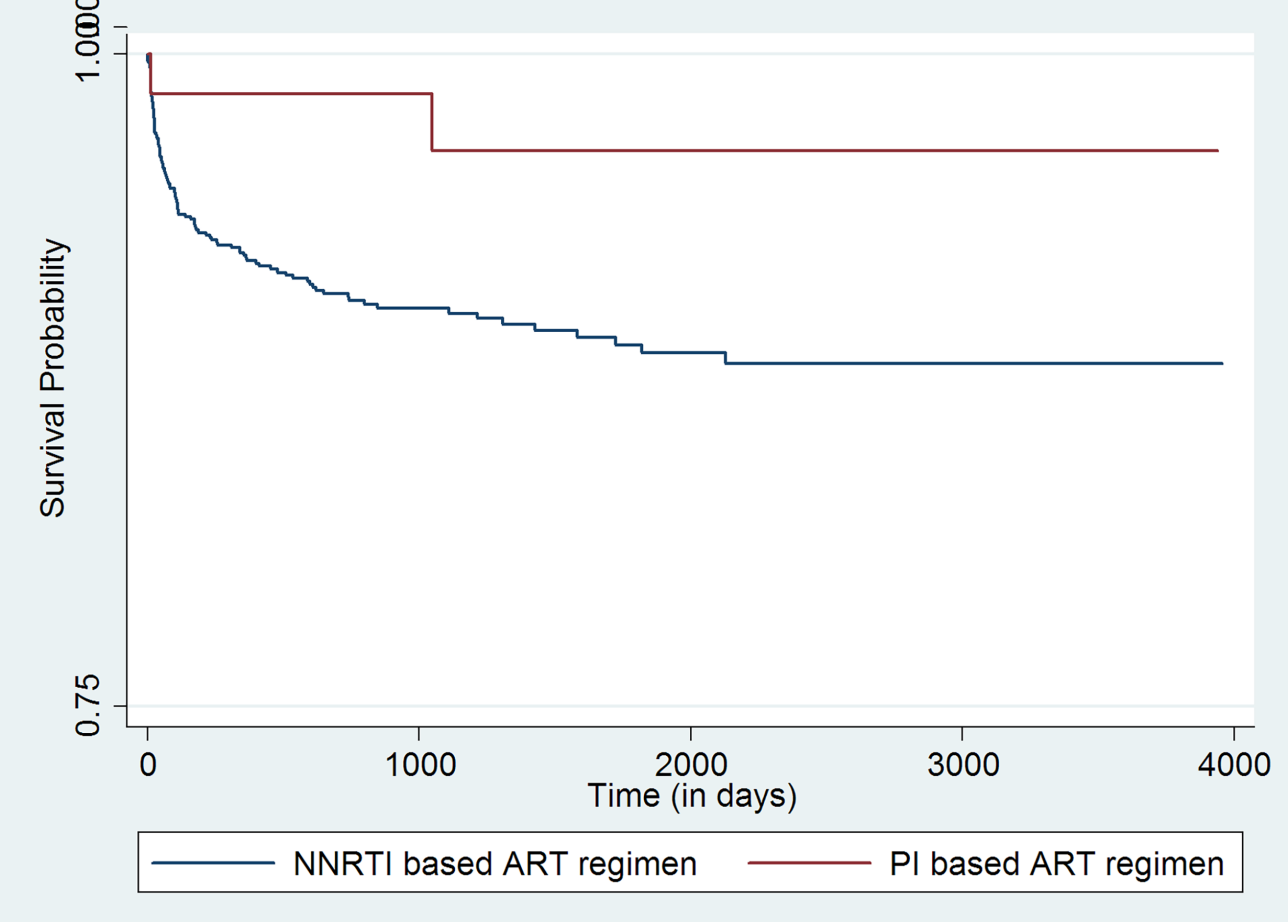
Unadjusted Kaplan-Meier survival curves for treatment outcome of HIV positive children who receiving ART in Mingalardon Specialist Hospital by regime of ART patients (2006 to 2016). Log rank test chi2 p-value: 0.0531.

Initial bivariate analysis using Kaplan-Meir survival curves (log rank test chi-square p-value <0.05) indicated age group, point of entry into ART care, CD4 cell count category, ART guideline being followed at the time of entry into the cohort, and the ART regimen (NNRTI-based regime or PI-based regime) that the child received at enrollment were statistically associated with adverse outcomes [**Figs (1–4)]**.

**Table 4** shows the unadjusted and adjusted hazard ratios of the association between demographic-clinical factors and adverse outcomes. On bivariate analysis, age group, WHO stage, point of entry into ART care, CD4 cell count, ART guideline at the time of enrollment, were statistically associated with adverse outcome. On multi-variable analysis point of entry into ART care, CD4 Cell count, ART guideline at the time of enrollment, the regimen that the child received at enrollment were statistically associated with adverse outcomes.

**Table 4:** Socio-demographic and clinical factors associated with adverse outcome of HIV positive children (age<15 years) [N = 1159] at Mingalardon Specialist Hospital from 2006 to 2016.

Variable	Number (N)	Adverse outcome							
		n	%	Unadjusted HR	95% CI	P-Value	Adjusted HR	95% CI	P-value
Age									
<1 years	83	14	16.9	2.31	(1.25–4.27)	0.007	1.8	(0.94–3.38)	0.076
1–5 years	397	36	9.1	1.14	(0.72–1.80)	0.563	1.4	(0.87–2.26)	0.158
5–10 years	482	40	8.3	Reference					
>10–15 years	197	22	11.2	1.47	(0.87–2.49)	0.145	1.4	(0.78–2.31)	0.272
Sex									
Male	610	58	9.5	0.93	(0.64–1.35)	0.710	0.9	(0.63–1.36)	0.73
Female	549	54	9.8	Reference					
Permanent Address									
Urban	965	88	9.1	Reference					
Rural	194	24	12.4	1.46	(0.92–2.32)	0.104	1.1	(0.67–1.79)	0.693
Entry point of care									
Out-patient	906	62	6.8	Reference					
In-patient	228	46	20.2	3.62	(2.45–5.34)	<0.001	2.6	(1.68–4.06)	<0.001
Others	18	1	5.6	0.76	(0.10–5.49)	0.788	0.7	(0.09–5.21)	0.729
Unknown	7	3	42.9	9.36	(2.93–29.87)	<0.001	7.1	(1.96–25.88)	0.003
WHO Staging									
Stage 1	231	12	5.2	Reference					
Stage 2	109	11	10.1	1.79	(0.79–4.07)	0.161	1.4	(0.59–3.26)	0.446
Stage 3	623	59	9.5	1.53	(0.82–2.87)	0.177	1.0	(0.47–2.18)	0.966
Stage 4	196	30	15.3	2.4	(1.22–4.73)	0.011	1.3	(0.53–2.91)	0.603
CD4 Count baseline									
<50 cells/mm3	199	34	17.1	2.52	(1.50–4.24)	<0.001	2.3	(1.27–4.06)	0.005
50–200 cells/mm3	246	24	9.8	1.45	(0.83–2.54)	0.191	1.3	(0.74–2.45)	0.326
201–350 cells/mm3	147	11	7.5	1.09	(0.54–2.23)	0.794	1.2	(0.58–2.48)	0.616
351–500 cells/mm3	120	5	4.2	0.6	(0.23–1.57)	0.299	0.7	(0.26–1.83)	0.467
>500 cells/mm3	384	26	6.8	Reference					
Missing	63	12	19.0	3.06	(1.54–6.10)	0.001	2.3	(1.09–4.84)	0.028
ART Guideline									
Guideline 2005	39	8	20.5	3.99	(1.54–10.31)	0.004	2.8	(0.99–8.02)	0.05
Guideline 2007	221	11	5.0	Reference					
Guideline 2011	477	59	12.4	2.97	(1.54–5.72)	0.001	2.1	(1.06–4.23)	0.033
Guideline 2014	422	34	8.1	2.54	(1.25–5.14)	0.009	1.8	(0.79–3.87)	0.166
TB at enrollment									
Yes	674	77	11.4	1.46	(0.97–2.18)	0.064	1.2	(0.66–1.93)	0.591
No	485	35	7.2	Reference					
Prophylaxis for OI									
Yes	960	94	9.8	0.99	(0.59–1.64)	0.978	1.1	(0.61–1.82)	0.843
No	199	18	9.0	Reference					
Regime of ART patients									
NNRTI based regime	1092	109	10.0	Reference					
PI based regime	67	3	4.5	0.27	(0.06–1.11)	0.093	0.2	(0.05–0.95)	0.043

HR = Hazard ratios; CD4 = CD4 +T lymphocyte count; ART = Anti-retroviral therapy; OI = Opportunistic infection; NNRTI = Non-nucleoside reverse-transcriptase inhibitors; PI = Protease Inhibitor.

Children enrolled into the ART program through the in-patient care department or those whose referral to ART care center status was ‘unknown’ had higher hazards of adverse outcome when compared to children who entered into ART care after referral from the out-patient department. Within the CD4 count categories, those having CD4 cell count less than 50 cells/mm^3^ or those children whose CD4 Cell count wasn’t available had a higher hazard of adverse outcome when compared to those with CD4 cell count >500 cells/mm^3^. Those who were enrolled at the time of 2005 ART guideline, 2011 or 2014 ART guideline had higher hazards of adverse outcome when compared with children who were enrolled at the time of 2007 ART guidelines. Children who received Protease inhibitor based ART regimen at enrollment had lower hazards of adverse outcome when compared to children who received NNRTI based regime.

## Discussion

In children enrolled for ART care at Mingalardon specialist hospital from 2006 to 2016, ~10% had an adverse outcome by 31^st^ March, 2017. To our knowledge, this is the first manuscript from Myanmar to describe the association between demographic-clinical characteristics at enrollment into ART care and adverse outcomes.

The major strengths of our study includes a large cohort of children that represents ~15% of 7298 children ever enrolled into ART care in Myanmar till 2016, with nearly 1.5 million person-days of follow-up time period data. In addition, we did not exclude any children. More than 95% of the children had data on all the study variables. Therefore, our study findings are likely to be robust.

There are three major limitations of this study: First, we used routinely collected programme data for our analysis and therefore we are unable to rule out measurement/misclassification errors. As standard procedures are being followed for obtaining patient information and with relatively well defined supervision and monitoring systems, the measurement errors are likely to be minimal & random. Therefore, our study results are unlikely to be biased due to these errors. Second, our study reflects the survival of children on pediatric ART care and does not provide any information on the survival of these children when they move into adult ART care after attaining 15 years of age. Third, we did not have information on a couple of important clinical factors that are associated with adverse outcomes in other studies such as nutritional status and baseline HIV viral load at entry and therefore we are unable to report in our cohort. By taking this opportunity, we can advocate the program for routine viral load monitoring in Myanmar as soon as possible.

Apart from low CD4 count, the other factors that we found associated with adverse outcomes are to be considered as independent predictors of long term adverse outcomes at enrolment into ART care. We strongly believe that these factors are surrogates of some unmeasured patient or health system characteristic that led them to have an association with the adverse outcome. For instance, relatively higher hazards of adverse outcomes in children referred from in-patient department in comparison to those referred from outpatient department of the hospital, most likely indicates that these children had some serious life threatening illness. Similarly, relatively higher hazards of death among children who were enrolled during 2005, 2011 and 2014 guidelines when compared to children enrolled during 2007 guidelines likely reflects health system, patient factors prevalent at those time periods and should not be interpreted as the effectiveness of these guidelines.

In our setting we observed lower hazards of adverse outcome in children (n = 67) who were initiated on PI based ART regimen at enrollment when compared to NNRTI based regimen. The exact reasons why some of these children were initiated on PI based regimen is not documented in our records and therefore we are unable to provide this information. This could be due to the unmeasured differences in the children’s ART therapy intake history prior to enrolment into HIV care at our hospital which led to prescription of protease inhibitor-based regime. This should not be construed as evidence for protease inhibitor-based regimen being more efficacious than NNRTI based regimen. Identifying the real underlying causes that lead to better outcome in children initiated on PI based regimen when compared to NNRTI based regimen is a potential area for future research.

Apart from this, our study findings are consistent with the high cumulative survival probabilities reported in pediatric ART cohorts from our hospital (previously) and in other countries such as Cambodia, Malawi, Lesotho, Swaziland[11–13]. In addition, non-finding of statistically significant differences in the hazards of outcome in children of all age groups, gender, residing in urban or rural areas, with or without TB at enrollment likely indicates that the pediatric ART services are being implemented efficiently with good co-ordination between HIV and TB control programmes in providing services to co-infected patients.

There were a couple of findings that suggest possible areas for further improvement. First, a large number of children entered into the pediatric ART cohort after 1 year of age. Since most of them had acquired infection through their mothers, there should be more focus on preventing the mother to child transmission of HIV infection, early diagnosis of HIV infection in children at risk of mother to child transmission. Therefore strengthening prevention of mother to child transmission programme for early diagnosis of HIV infection in children may be required. Second, nearly half of the children had tuberculosis at diagnosis. This proportion is relatively high when compared to TB at enrolment seen in other countries indicating possibly delays in HIV diagnosis or delays in enrolling children on ART care without tuberculosis. The specific reasons for these delays need further evaluation.

Finally, it is commendable to note that pediatric ART center at this hospital has maintained electronic database of all children ever enrolled into pediatric ART care since its inception in 2005. This allowed us to use this database to conduct our study and assess long term survival in children. The maintenance of electronic database can act as a model for other similar centers in the world.

**In conclusion**, only 10% of the children ever enrolled into pediatric ART care had an adverse outcome (death or loss to follow-up) in the pediatric cohort with nearly 1.5 million person days of follow-up. This indicates good survival and/or good pediatric care programme implementation and this should continue. Of all the major demographic and clinical factors at enrollment, those associated with adverse outcomes included entry into ART care from in-patient department, CD4 Cell count <50, enrollment during 2005, 2011 and 2014 ART guideline periods. Identifying the underlying causes for these factors to be associated with adverse outcomes is a potential area for future research and this research is needed to identify the interventions for further improving the survival of children in ART care in Myanmar.
